# Successful transition from rhPTH(1-84) to palopegteriparatide in chronic hypoparathyroidism

**DOI:** 10.1210/jcemcr/luag048

**Published:** 2026-03-26

**Authors:** Isabella Chiardi, Pierpaolo Trimboli

**Affiliations:** Department of Internal Medicine and Therapeutics, University of Pavia, Pavia 27100, Italy; Thyroid Unit, Clinic for Endocrinology and Diabetology, Ente Ospedaliero Cantonale (EOC), Bellinzona 6500, Switzerland; Faculty of Biomedical Sciences, Università Della Svizzera Italiana, Lugano 6900, Switzerland

**Keywords:** hypoparathyroidism, rhPTH (1-84), TransCon PTH, palopegteriparatide, Yorvipath, therapy transition

## Abstract

Hypoparathyroidism management has advanced in recent decades, particularly for patients unresponsive to conventional therapy. Several parathyroid hormone–based agents have been developed to better imitate physiologic hormone replacement. Recombinant human parathyroid hormone (1-84) [rhPTH(1-84), Natpar®] was approved as an adjunct to calcium and vitamin D for chronic hypoparathyroidism but was later withdrawn, with global production ceasing in 2024. Palopegteriparatide (Yorvipath®; TransCon PTH), a long-acting parathyroid hormone (1-34) prodrug recently approved, represents a new-generation replacement therapy. Transitioning patients from rhPTH(1-84) to palopegteriparatide is increasingly relevant but remains scarcely documented, with only a single real-world report available. We describe a patient with chronic postsurgical hypoparathyroidism after total thyroidectomy who continued to experience symptoms despite conventional therapy. rhPTH(1-84) initially improved symptoms and stabilized calcium levels, but additional supplementation became necessary over time. In September 2024, ahead of rhPTH(1-84) withdrawal, therapy was switched to palopegteriparatide, allowing gradual dose titration and complete withdrawal of calcium and magnesium supplements. The patient maintained stable biochemical values and reported improved well-being, with no adverse events. This case supports the effective transition from rhPTH(1-84) to palopegteriparatide, restoring biochemical stability and independence from conventional therapy.

## Introduction

Hypoparathyroidism is an endocrine disorder characterized by insufficient or absent secretion of parathyroid hormone (PTH) resulting in hypocalcemia. Chronic hypoparathyroidism (HypoPT) remains one of the few endocrine diseases for which hormone replacement therapy has not yet become standard first-line treatment. HypoPT adversely affects health-related quality of life, physical functioning, and psychological well-being, irrespective of serum calcium levels [1, 2]. Conventionally, HypoPT patients have been managed with oral calcium and active vitamin D to only correct hypocalcemia. However, long-term use of these agents is associated with complications such as nephrolithiasis, nephrocalcinosis, extra-skeletal calcifications, and chronic kidney disease [3]. Another limitation of conventional therapy is the difficulty in controlling serum phosphate levels resulting in hyperphosphatemia and/or an elevated calcium–phosphate product. Furthermore, conventional treatment fails to restore normal bone remodeling, leaving patients with the distinctive low-turnover skeletal phenotype and abnormal bone microarchitecture characteristic of HypoPT [4]. In addition, these patients have risk of hospitalization and other comorbidity higher than general population [5-7].

Over the past 2 decades, PTH-based therapies have been developed to more closely replicate physiologic hormone replacement. These include recombinant human PTH (1-84) [rhPTH(1-84), Natpar®], teriparatide [rhPTH(1-34)], and the PTH-related peptide analog abaloparatide. Of these, rhPTH(1-84) was the only previously approved as an adjunct to calcium and vitamin D for chronic HypoPT, but it was withdrawn from the market and its global production ceased in 2024 [8]. Teriparatide and abaloparatide, though approved for osteoporosis, are not formally indicated for HypoPT, despite some published reports of off-label use [9-11]. Table 1 summarizes the main differences among the PTH-based therapies developed.

**Table 1:** Summary of key differences among PTH-based therapies with respect to regulatory status, dosage, and clinical availability

Parameter	Palopegteriparatide	rhPTH(1-84)	Teriparatide
Approved for treatment	Yes	Yes (conditional)	No
Label	Indicated for the treatment as replacement therapy	Indicated as adjunctive treatment in patients not controlled with standard therapy alone	N.A.
Orphan drug status	Yes	Yes	N.A.
Dosage	6-60 mcg (3-mcg increment)	25, 50, 75 or 100 mcg/day	20 mcg/day
Current availability	Approved by FDA, EMA and PMDA	Withdrawn	Off label use

Abbreviations: EMA, European Medicines Agency; FDA, Food and Drug Administration; PMDA, Pharmaceuticals and Medical Devices Agency; rhPTH, recombinant human parathyroid hormone.

Palopegteriparatide (Yorvipath®; TransCon PTH) represents a new-generation PTH conceived for replacement use. It is a long-acting prodrug of PTH(1-34) designed for once-daily subcutaneous administration, providing continuous, physiologic PTH exposure over 24 hours. The released active hormone has an estimated half-life of about 60 hours [12, 13]. In November 2023, August 2024, and 2025, the European Medicines Agency, the US Food and Drug Administration, and the Pharmaceuticals and Medical Devices Agency, respectively, approved palopegteriparatide injection as the first and only treatment for adults with HypoPT [14-16]. Results from recent phase II and III clinical studies have shown that palopegteriparatide can overcome several of the limitations observed with conventional HypoPT therapy and other PTH-based treatments [17-19].

The present case report describes the clinical experience of transitioning a patient with chronic postsurgical hypoparathyroidism from rhPTH(1-84), which is no longer available, to palopegteriparatide. Sparse data in this setting of HypoPT patients exists, and insights emerge from the present case report.

## Case presentation

We report the case of a 41-year-old female with a multinodular goiter known since 2005 and preserved thyroid function. She had normal body weight and no relevant family history or other endocrine disorders. In April 2016, she underwent total thyroidectomy due to progressive neck compressive symptoms. Two days postoperatively, she developed a tetanic episode (corrected calcium: 7.4 mg/dL [SI: 1.84 mmol/L]; reference range, 8.6-10.2 mg/dL [SI: 2.15-2.55 mmol/L]) requiring intravenous calcium gluconate. She was then discharged without calcium supplementation but experienced persistent neuromuscular symptoms over the following year. Physical examination showed positive Trousseau and Chvostek signs. By May 2017 she initiated oral calcium supplementation. Despite treatment with oral calcium and calcitriol, the patient continued to experience symptoms and biochemical instability despite high doses. Anxiety represented the major symptom since the HypoPT occurred with no amelioration with treatment.

## Diagnostic assessment

The diagnosis of postsurgical hypoparathyroidism was confirmed based on persistent hypocalcemia and undetectable PTH levels. No additional imaging studies were performed.

## Treatment

In February 2018, rhPTH 1-84 was initiated at 50 mcg/day, resulting in rapid clinical improvement and complete discontinuation of calcium and calcitriol. This regimen ensured stable disease control for several years. However, in June 2024, the patient experienced a hypocalcemic episode requiring oral calcium. Since then, she required 400 mg/day of calcium carbonate but reported adverse effects including vomiting and decreased quality of life.

The patient was first seen at Thyroid Unit of Ente Ospedaliero Cantonale in Lugano in July 2024 for consultation. She complained of persistent symptoms such as difficulty with daily activities and vomiting following calcium intake. Ongoing therapy consisted of rhPTH 1-84 50 mcg daily, levothyroxine 100 mcg daily, calcium carbonate 400 mg daily, and vitamin D and magnesium supplementation. Laboratory values at this time were: calcium 10.0 mg/dL (SI: 2.49 mmol/L), phosphorus 2.0 mg/dL (SI: 0.66 mmol/L; reference range, 2.5-4.5 mg/dL [SI: 0.81-1.45 mmol/L]), and albumin 4.9 g/dL (SI: 49 g/L; reference range 3.5-5.0 g/dL [SI: 35-50 g/L]). In light of the planned discontinuation of rhPTH(1-84) commercialization in Europe as of December, the patient sought information regarding potential alternative treatment options.

On September 3, 2024, palopegteriparatide 18 mcg daily was initiated, with discontinuation of rhPTH 1-84 while maintaining calcium carbonate, magnesium, and vitamin D supplementation. Laboratory values were: calcium 10.8 mg/dL (SI: 2.71 mmol/L), phosphorus 2.3 mg/dL (SI: 0.75 mmol/L), and albumin 4.6 g/dL (SI: 46 g/L). Subsequently, calcium and vitamin D supplementation were successfully discontinued after 1 week of treatment, with stable biochemical control (calcium 10.5 mg/dL [SI: 2.63 mmol/L], phosphorus 3.0 mg/dL [SI: 0.96 mmol/L]). Also, magnesium supplementation had been stopped after ∼1 month of treatment with no biochemical changes observed.

Stepwise dose reductions of palopegteriparatide were then attempted. At 15 mcg/day, calcium was 10.3 mg/dL (SI: 2.58 mmol/L) and phosphorus was 3.0 mg/dL (SI: 0.96 mmol/L); at 12 mcg/day, calcium was 10.0 mg/dL (SI: 2.50 mmol/L) and phosphorus was 3.0 mg/dL (SI: 0.98 mmol/L).

Table 2 summarizes the biochemical values over time with different ongoing therapies.

**Table 2:** Summary of serum calcium, phosphorus, and albumin levels measured at different time points under various therapeutic regimens

Ongoing therapy	rhPTH 1-84 50 mcg	Palopegteriparatide 18 mcg	Palopegteriparatide 18 mcg	Palopegteriparatide 15 mcg	Palopegteriparatide 15 mcg	Palopegteriparatide 12 mcg
	Calcium 400 mg	Calcium 400 mg	Magnesium suppl.	Magnesium suppl.		
	Magnesium and vitamin D suppl.	Magnesium and vitamin D suppl.				
Date	11/07/2024	10/09/2024	17/09/2024	01/10/2024	11/10/2024	21/11/2024
Calcium (2.15-2.55 mmol/L; 8.6-10.2 mg/dL)	2.49 mmol/L (9.96 mg/dL)	2.71 mmol/L (10.84 mg/dL)	2.63 mmol/L (10.52 mg/dL)	2.65 mmol/L (10.60 mg/dL)	2.58 mmol/L (10.32 mg/dL)	2.50 mmol/L (10.00 mg/dL)
Phosphorus (0.81-1.45 mmol/L; 2.5-4.5 mg/dL)	0.66 mmol/L (2.04 mg/dL)	0.75 mmol/L (2.32 mg/dL)	0.96 mmol/L (2.97 mg/dL)	0.93 mmol/L (2.88 mg/dL)	0.96 mmol/L (2.97 mg/dL)	0.98 mmol/L (3.03 mg/dL)
Albumin (35-50 g/L; 3.5-5.0 g/dL)	49 g/L	46 g/L	47 g/L	48 g/L	47 g/L	47 g/L
	(4.9 g/dL)	(4.6 g/dL)	(4.7 g/dL)	(4.8 g/dL)	(4.7 g/dL)	(4.7 g/dL)

## Outcome and follow-up

The patient achieved stable biochemical control with calcium and phosphorus levels within the reference range while maintained on palopegteriparatide 12 mcg/day monotherapy following a gradual dose reduction from 18 mcg/day over approximately 2.5 months. No further hypocalcemic episodes occurred, and symptoms improved significantly. At the last follow-up, she remained clinically stable, with normalization of quality of life and no requirement for calcium, vitamin D, or magnesium supplementation. Anxiety was substantially resolved.

## Discussion

According to the 2022 Guidelines on the Evaluation and Management of Hypoparathyroidism [20], PTH-based therapy is recommended for patients with HypoPT inadequately controlled on or intolerant to conventional therapy, including those with symptomatic hypocalcemia, hypercalciuria, hyperphosphatemia, poor quality of life, or evidence of renal insufficiency. In fact, chronic hypoparathyroidism is challenging to manage, particularly in patients who remain symptomatic despite conventional therapy. rhPTH(1-84) represented a major therapeutic advance but was withdrawn from the market [8], creating a treatment gap for patients relying on PTH replacement to maintain metabolic and symptomatic stability.

Palopegteriparatide was developed to provide more physiologic and sustained PTH exposure, resulting in improved calcium-phosphate homeostasis and reduced need for oral supplements. Its clinical development is supported by the PaTH Forward phase II [21] and PaTHway phase III trials [17, 19], which demonstrated efficacy, safety, and improved health-related quality of life, skeletal dynamics, and renal function [17-19, 22]. Compared to previous PTH analogs, palopegteriparatide consistently improves quality of life and kidney function while allowing full independence from conventional therapy [23].

The study by Graham et al [24]. evaluated the real-world impact of palopegteriparatide on clinical outcomes in adults with hypoparathyroidism. The analysis included 123 patients, of whom 62 (50.4%) transitioned directly from short-acting PTH or PTH-related protein therapy to palopegteriparatide; among these, 35 (56.5%) had previously received rhPTH (1-84). Although subgroup data for rhPTH (1-84) were not reported separately, combined results for patients switching from short-acting PTH therapies (teriparatide + rhPTH 1-84) showed that mean serum calcium levels remained within the reference range throughout treatment. Rates of hypocalcemia (13% vs 10%) and hypercalcemia (9% vs 10%) were comparable to those observed in the PaTHway trial. Notably, a direct switch without overlap between prior PTH therapy and palopegteriparatide was generally well tolerated.

Siggelkow et al [25]. reported the first clinical data on transitioning adults with hypoparathyroidism from rhPTH (1-84) to palopegteriparatide with individualized dose adjustment. Forty patients initiated treatment with 18 mcg daily; within the first month, 80% required titration to maintain normocalcemia—reductions were made in 38% and increases in 43% of cases. Dose adjustments were mainly guided by serum calcium, and prior rhPTH (1-84) dose correlated with the final palopegteriparatide requirement. Most adverse events were mild or moderate. Hypercalcemia occurred more often in patients previously on lower rhPTH (1-84) doses, while hypocalcemia was seen in those on higher doses (>100 mcg). The authors suggest starting palopegteriparatide at 15, 18, or 21 mcg for patients previously on <50, 50-75, or >75 mcg rhPTH (1-84), respectively, with close calcium monitoring. This study provides essential real-world guidance indicating that switching from rhPTH (1-84) to palopegteriparatide is safe, feasible, and well tolerated.

Our present case illustrates a successful transition from rhPTH (1-84) to palopegteriparatide. Following the switch, and the gradual step-down of the palopegteriparatide dose to 12 mcg/day, the patient achieved complete independence from calcium, vitamin D, and magnesium supplementation, with normalization of biochemical parameters, improved well-being, and no adverse events. This case here reported contributes to the limited clinical data currently available regarding the use of palopegteriparatide in patients previously treated with rhPTH (1-84). In this regard, it should be emphasized that the patient had maintained overall clinical stability while receiving rhPTH (1-84) and developed hypocalcemia only after years of treatment. This outcome is not unexpected, as a reduced or altered response to rhPTH(1-84) over time has been previously reported and may be attributable to several mechanisms, including the development of anti-PTH antibodies. Following the switch to palopegteriparatide, the patient clinical symptoms resolved despite unchanged serum calcium levels. Several hypotheses may be proposed to explain these findings from both pharmacokinetic and pharmacodynamic perspectives. In particular, a more physiological pattern of PTH exposure may enhance calcium homeostasis at peripheral target tissues, in particular the receptors of central and peripheral nervous systems. This results in improved clinical stability even in the absence of measurable changes in circulating calcium concentrations. Further long-term studies and real-world evidence are needed to define optimal dosing strategies and to confirm the durability of clinical benefits over time.

## Learning points

- Transition from rhPTH (1-84) to palopegteriparatide is feasible, safe, and well tolerated.
- Individualized dose titration is needed to maintain normocalcemia and minimize the risk of hypo- or hypercalcemia.
- Effective transition can allow patients’ independence from calcium, vitamin D, and magnesium supplementation.

## Contributors

P.T. was involved in the diagnosis and management of the patient. I.C. participated in critical revision of the case and wrote the first draft of the manuscript. P.T. reviewed and edited the manuscript. All authors reviewed and approved the final draft.

## Data Availability

Original data generated and analyzed for this case report are included in this published article.

## Abbreviations

EMA: European Medicines Agency
FDA: Food and Drug Administration
HypoPT: hypoparathyroidism
PMDA: Pharmaceuticals and Medical Devices Agency
PTH: parathyroid hormone
rhPTH(1–34): recombinant human parathyroid hormone (1–34)
rhPTH(1–84): recombinant human parathyroid hormone (1–84)
SI: International System of Units
TransCon PTH: transCon parathyroid hormone

## References

[1] Bilezikian JP, Khan A, Potts JT Jr, et al Hypoparathyroidism in the adult: epidemiology, diagnosis, pathophysiology, target-organ involvement, treatment, and challenges for future research. J Bone Miner Res. 2011;26(10):2317‐2337. 21812031 10.1002/jbmr.483 PMC3405491

[2] Kontogeorgos G, Mamasoula Z, Krantz E, Trimpou P, Landin-Wilhelmsen K, Laine CM. Low health-related quality of life in Hypoparathyroidism and need for PTH analog. Endocr Connect. 2022;11(1):e210379. 34825891 10.1530/EC-21-0379 PMC8789022

[3] Ketteler M, Chen K, Gosmanova EO, et al Risk of Nephrolithiasis and Nephrocalcinosis in patients with chronic Hypoparathyroidism: a retrospective cohort study. Adv Ther. 2021;38(4):1946‐1957. 33704680 10.1007/s12325-021-01649-2 PMC8004511

[4] Marini F, Giusti F, Weiss B, et al Bone status in patients with chronic Hypoparathyroidism: results from the Italian HypoparaNET database. Aging Clin Exp Res. 2025;37(1):230. 40702385 10.1007/s40520-025-03139-9 PMC12287157

[5] Underbjerg L, Sikjaer T, Mosekilde L, Rejnmark L. Cardiovascular and renal complications to postsurgical Hypoparathyroidism: a Danish nationwide controlled historic follow-up study. J Bone Miner Res. 2013;28(11):2277‐2285. 23661265 10.1002/jbmr.1979

[6] Underbjerg L, Sikjaer T, Mosekilde L, Rejnmark L. Postsurgical Hypoparathyroidism–risk of fractures, psychiatric diseases, cancer, cataract, and infections. J Bone Miner Res. 2014;29(11):2504‐2510. 24806578 10.1002/jbmr.2273

[7] Underbjerg L, Sikjaer T, Mosekilde L, Rejnmark L. The epidemiology of nonsurgical Hypoparathyroidism in Denmark: a nationwide case finding study. J Bone Miner Res. 2015;30(9):1738‐1744. 25753591 10.1002/jbmr.2501

[8] Takeda . Takeda to discontinue manufacturing of NATPAR®/NATPARA® for patients with hypoparathyroidism at the end of 2024. 2022. Accessed November 2, 2025. https://www.takeda.com/en-us/newsroom/statements/2022/takeda-to-discontinue-manufacturing-of-natpar-natpara/

[9] Gamarra E, Retta F, Lucatello B, et al Continuous subcutaneous rhPTH infusion for managing difficult chronic Hypoparathyroidism. A systematic review. Endocrine. 2023;81(2):194‐205. 37017857 10.1007/s12020-023-03355-1

[10] Saraiva M, Chaves AC, Assuncao G, Saraiva J, Carvalho R. Continuous teriparatide treatment in chronic Hypoparathyroidism: a case report. Am J Case Rep. 2021;22:e931739. 34389697 10.12659/AJCR.931739 PMC8370137

[11] Chamseddin R, Mathew A, Alzohaili O. Abstract #1407986: abaloparatide administration through the simplicity patch: a therapeutic option for refractory Hypoparathyroidism post-thyroidectomy. Endocr Pract. 2023;29(5):S57‐S58.

[12] Karpf DB, Pihl S, Mourya S, et al A randomized double-blind placebo-controlled first-in-human phase 1 trial of TransCon PTH in healthy adults. J Bone Miner Res. 2020;35(8):1430‐1440. 32212275 10.1002/jbmr.4016 PMC9328939

[13] Holten-Andersen L, Pihl S, Rasmussen CE, et al Design and preclinical development of TransCon PTH, an investigational sustained-release PTH replacement therapy for Hypoparathyroidism. J Bone Miner Res. 2019;34(11):2075‐2086. 31291476 10.1002/jbmr.3824

[14] Ascendis Pharma . FDA approves YORVIPATH® (palopegteriparatide) as the first and only treatment for hypoparathyroidism in adults. Press release. Accessed August 12, 2024. https://investors.ascendispharma.com/news-releases/news-release-details/fda-approves-yorvipathr-palopegteriparatide-first-and-only

[15] Ascendis Pharma . Ascendis Pharma announces European Commission approval of YORVIPATH® (palopegteriparatide) for the treatment of adults with chronic hypoparathyroidism. Press release. Accessed November 20, 2023. https://investors.ascendispharma.com/news-releases/news-release-details/ascendis-pharma-announces-european-commission-approval

[16] Teijin Pharma Limited . Teijin Pharma receives manufacturing and marketing approval in Japan for YORVIPATH® as the treatment for hypoparathyroidism. Press release. Accessed August 25, 2025. https://www.teijin.com/news/2025/08/25/20250825_01.pdf

[17] Clarke BL, Khan AA, Rubin MR, et al Efficacy and safety of TransCon PTH in adults with Hypoparathyroidism: 52-week results from the phase 3 PaTHway trial. J Clin Endocrinol Metab. 2025;110(4):951‐960. 39376010 10.1210/clinem/dgae693 PMC11913112

[18] Rejnmark L, Gosmanova EO, Khan AA, et al Palopegteriparatide treatment improves renal function in adults with chronic Hypoparathyroidism: 1-year results from the phase 3 PaTHway trial. Adv Ther. 2024;41(6):2500‐2518. 38691316 10.1007/s12325-024-02843-8 PMC11133178

[19] Khan AA, Rubin MR, Schwarz P, et al Efficacy and safety of parathyroid hormone replacement with TransCon PTH in Hypoparathyroidism: 26-week results from the phase 3 PaTHway trial. J Bone Miner Res. 2023;38(1):14‐25. 36271471 10.1002/jbmr.4726 PMC10099823

[20] Khan AA, Bilezikian JP, Brandi ML, Clarke BL, Potts JJ, Mannstadt M. The second international workshop on the evaluation and management of hypoparathyroidism. J Bone Miner Res. 2022;37(12):2566‐2567. 36375811 10.1002/jbmr.4671

[21] Khan AA, Rejnmark L, Rubin M, et al PaTH forward: a randomized, double-blind, placebo-controlled phase 2 trial of TransCon PTH in adult Hypoparathyroidism. J Clin Endocrinol Metab. 2022;107(1):E372‐E385. 34347093 10.1210/clinem/dgab577 PMC8684498

[22] Schwarz P, Rejnmark L, Gosmanova EO, et al Sustained improvement in renal function with palopegteriparatide in adults with chronic hypoparathyroidism: 2-year results from the phase 3 PaTHway trial. Oral Presentation at European Congress of Endocrinology; May 11–14, 2024, Stockholm, Sweden. European Society of Endocrinology. 10.1093/jbmr/zjag085 PMC13525197 42166177

[23] Palermo A, Naciu AM, Donovan YKT, Tabacco G, Zavatta G. PTH substitution therapy for chronic Hypoparathyroidism: PTH 1–84 and palopegteriparatide. Curr Osteoporos Rep. 2025;23(1):12. 39987371 10.1007/s11914-025-00905-6

[24] Graham T, Shoback DM, Abbott L, et al Early U.S. Real-world treatment patterns and outcomes in palopegteriparatide treatment for patients with Hypoparathyroidism. Endocr Pract. 2025;31(12):1568‐1575. 40846304 10.1016/j.eprac.2025.08.008

[25] Siggelkow H, Peschke KA, Tsourdi E, et al Switch from rhPTH1-84 to TransCon PTH with individual dose adjustment in adult Hypoparathyroidism—4-week results. J Endocr Soc. 2025;9(9):bvaf113. 40709359 10.1210/jendso/bvaf113 PMC12287600

